# Stability-Indicating HPLC Assay for Determination of Idebenone in Pharmaceutical Forms

**DOI:** 10.1155/2015/835986

**Published:** 2015-10-29

**Authors:** Sonoube Kombath, Issa-Bella Balde, Sandra Carret, Sofiane Kabiche, Salvatore Cisternino, Jean-Eudes Fontan, Joël Schlatter

**Affiliations:** Service Pharmacie, Hôpital Jean-Verdier, Assistance Publique des Hôpitaux de Paris (AP-HP), Hôpitaux Universitaires de Paris-Seine-Saint-Denis, avenue du 14 Juillet, 93140 Bondy, France

## Abstract

A stability-indicating method was validated for the determination in pharmaceutical forms of idebenone a coenzyme Q10-like compound. The assay was achieved by liquid chromatography analysis using a reversed-phase C18 column and a detector set at 480 nm. The optimized mobile phase consisted of isocratic flow rate at 1.0 mL/min for 3 min with methanol. The linearity of the assay was demonstrated in the range of 3.0 to 8.0 mg/mL with a correlation coefficient *r* ^2^ > 0.998. The limits of detection and quantification were 0.03 and 0.05 mg/mL, respectively. The intraday and interday precisions were less than 1.0%. Accuracy of the method ranged from 98.6 to 101.5% with RSD < 0.6%. Specificity of the assay showed no interference from tablets components and breakdown products formed by alkaline, acidic, oxidative, sunlight, and high temperature conditions. This method allows accurate and reliable determination of idebenone for drug stability assay in pharmaceutical studies.

## 1. Introduction

**Figure 1:**
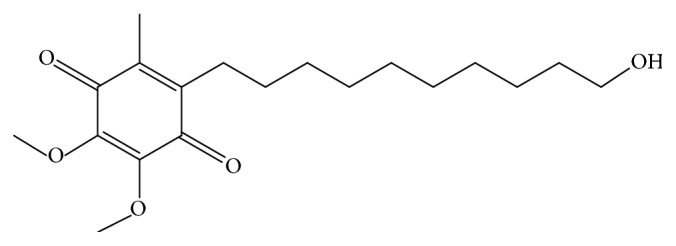
Chemical structure of idebenone.

Idebenone (2-(10-hydroxydecyl)-5,6-dimethoxy-3-methyl-cyclohexa-2,5-diene-1,4-dione) (Figure 1) is a synthetic analogue of ubiquinone (coenzyme Q10), a known physiological mitochondria's membrane compound. Idebenone appears to act as a coenzyme Q10-like compound exhibiting similarly antioxidant and electron chain transport properties involved in the generation of ATP [1, 2]. Idebenone has been reported to improve energetic metabolism deficiencies and to relieve neurologic symptoms [3, 4]. Initially developed for the treatment of cognitive disturbances and Alzheimer's disease, idebenone is approved as an orphan drug by the Food Drug and Administration (FDA) and European agencies for the treatments of Friedreich's ataxia, Leber's hereditary optic neuropathy, or Duchenne muscular dystrophy [5–7]. Reported methods for the determination of ubiquinones are available in biological fluids by a reverse phase HPLC with a step extraction procedure, UV detection, and a run time of about 20 min [8, 9]. We report the first reverse phase HPLC stability-indicating method for assessing idebenone in pharmaceutical forms using a visible detection, direct injection, and a run time of 3 min. According to the International Conference on Harmonisation (ICH) stability guideline, the method was validated by forced oxidative, alkaline, and acidic degradations studies [10].

## 2. Material and Method

### 2.1. Reagents and Chemicals

Idebenone pharmaceutical-quality grade powder was supplied by Inresa (Bartenheim, France, lot PR140151B). The pharmaceutical dosage tablets were purchased from Intsel Chimos (Mnesis, St. Cloud, France). HPLC-grade methanol was provided by Merck KGaA (Lichrosolv, Darmstadt, Germany).

### 2.2. Equipment

A Dionex Ultimate 3000 system (Thermo Scientific, Villebon sur Yvette, France) was used for the assay. This HPLC system contained an integrated solvent and degasser SRD-3200, an analytical pump HPG-3200SD, a thermostated autosampler WPS-3000TSL, a thermostated column compartment TCC-3000SD, and a diode array detector MWD-3000. Data acquisition was carried out using in-line Chromeleon software (v6.80 SP2) (Thermo Scientific).

### 2.3. Chromatographic Conditions

The eluent was monitored at 480 nm. Chromatographic separation was achieved at 25°C using a Nova-Pak C18 column (Waters, Guyancourt, France) with 4 *μ*m particle size, 4.6 mm internal diameter, and 150 mm length. The mobile phase of 100% methanol was pumped at a flow rate of 1.0 mL/min. The injection sample volume was set at 25 *μ*L. The method run time was 3 min and all experiments were in triplicate.

### 2.4. Preparation of Stock Solution

Idebenone stock solution (100 mg/mL) was prepared by accurately weighing 10 g and transferring to 100 mL volumetric flask. Volume was made up to the mark with methanol. The stock solution was stored at 2–8°C during 5 days.

### 2.5. Sample Preparation

Two idebenone 45 mg tablets were crushed to a fine powder in a glass mortar and 5 mL of methanol was added. The suspension was transferred to a 10 mL volumetric flask and made up to 10 mL with methanol to achieve a final idebenone 9 mg/mL concentration. The recovery of idebenone from tablets was 100.4 ± 0.3%.

### 2.6. Preparation of Calibration Standards and Quality Control Samples

Calibration standards at 3.0, 4.0, 5.0, 6.0, 7.0, and 8.0 mg/mL were freshly prepared using the stock solution. Quality control solutions at 3.5, 4.5, and 6.5 mg/mL were prepared extemporaneously.

### 2.7. Analytical Method Validation

Validation studies were performed according to the ICH guidelines [10]. The method was validated for specificity, linearity, limit of detection (LOD), limit of quantification (LOQ), precision, accuracy, robustness, and the stability-indicating capability.

The specificity was determined by analyzing the chromatograms of tablets in comparison with those obtained for idebenone standard solution aiming at confirming that none of the ingredients interfere with the quantitation of the drug.

The linearity was determined by a least-square linear regression routine using the compound peak area and concentration of the working standard solutions prepared at six concentration levels (3.0, 4.0, 5.0, 6.0, 7.0, and 8.0 mg/mL). Six replicates of each concentration were independently prepared and injected in triplicate into the chromatograph. The method was evaluated by determination of the correlation coefficient and intercept values according to the ICH guidelines.

LOD and LOQ were determined by calibration curve method. Solutions of idebenone were prepared in linearity range and injected in triplicate. Average peak area of three analyses was plotted against concentration. LOD and LOQ were expressed as 3.3 × Syx/*b* and 10.0 × Syx/*b*, respectively, where Syx is residual variance due to regression and *b* is the mean slope of the linear regression curves.

The precision was assessed at intraday and interday precision. The intraday precision was determined by measuring quality control samples of 3.5, 4.5, and 6.5 mg/mL of idebenone, injected six times on the same day. The intermediate (interday) precision was estimated by injecting quality control samples prepared at the same concentrations on three different days by different operators. Results were reported in terms of relative standard deviation (RSD).

The accuracy was investigated by using the standard addition method at different levels: 20, 40, 80, 100, and 120%. The mean recovery of idebenone of the target concentration (4 mg/mL) was calculated and accepted with 100 ± 2%.

To determine the robustness, experimental HPLC conditions were purposely modified to check the reproducibility of the method. The evaluation of robustness was based on RSD values obtained by changing analytical setting such as isocratic flow rate (1.1 to 1.3 mL/min), wavelength detection (481 to 483 nm), temperature of analytical column (23 to 30°C), and composition of mobile phase (1 and 2% deionised water).

### 2.8. Forced Degradation Study

Forced degradation studies were carried out to provide some information about the drug stability and to validate the specificity of the idebenone quantification of the assay. The standard solution of idebenone was exposed to accelerated degradation by alkaline, acid, and oxidative and direct exposure to sunlight conditions.

To perform the alkaline hydrolysis, 2 mL of idebenone stock solution (100 mg/mL) was mixed in a 4 mL tube with 800 *μ*L of 1 N sodium hydroxide (NaOH) for 1 h and maintained in the dark at 50°C. After the reaction time, this solution was cooled at room temperature and neutralized with 1 N hydrochloric acid (HCl). The solution was completed with methanol to reach a targeted concentration of 5 mg/mL before injection into the HPLC system. The same procedure was used for the acid hydrolysis using 1 N HCl in sample preparation and 1 N NaOH for neutralization before HPLC assay.

To investigate oxidative degradation, 2 mL of idebenone stock solution was mixed in a 4 mL tube with 1 mL of 3% hydrogen peroxide for 48 h at 50°C in the dark. This solution was completed with methanol to reach a targeted concentration of 5 mg/mL before injection into the HPLC system.

For the photolytic degradation, 2 mL of idebenone stock solution was transferred in a 4 mL tube and directly exposed to sunlight for 5 days. The solution was completed with methanol to reach a targeted concentration of 5 mg/mL.

## 3. Results and Discussion

### 3.1. Analytical Method

**Figure 2:**
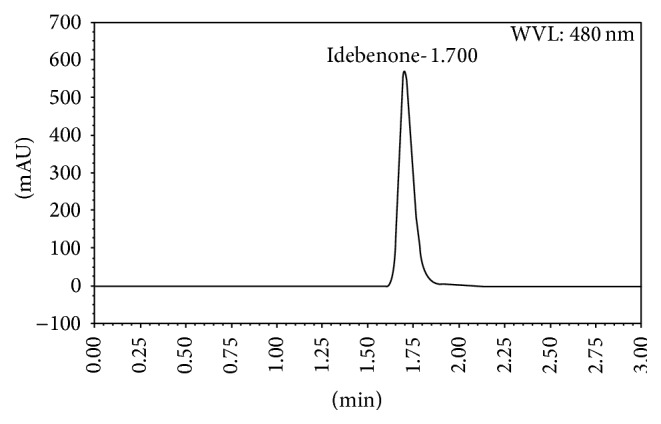
Representative HPLC chromatogram of idebenone standard (4 mg/mL) in methanol mobile phase; flow rate: 1.0 mL/min; detection wavelength: 480 nm; column temperature: 25 ± 3°C; and injection volume: 25 *μ*L.

To achieve a quick and effective separation, idebenone was injected into different mobile phase solutions mixing deionised water and methanol at different proportions. Due to the insolubility of idebenone in water, the various assays demonstrated that the proportion of 100% methanol was more appropriate for the assay and an idebenone elution was observed at 1.70 min. Column temperature was found to be not a critical factor of the analysis. The optimum drug absorbance was obtained at 480 nm, as there was no interference from excipients present in commercial tablets. A typical chromatogram obtained with the present method is shown in Figure 2.

### 3.2. Method Validation

The proposed method was validated by determining its performance characteristics regarding specificity, linearity, LOD, LOQ, accuracy, precision, and robustness.

#### 3.2.1. Specificity

**Figure 3:**
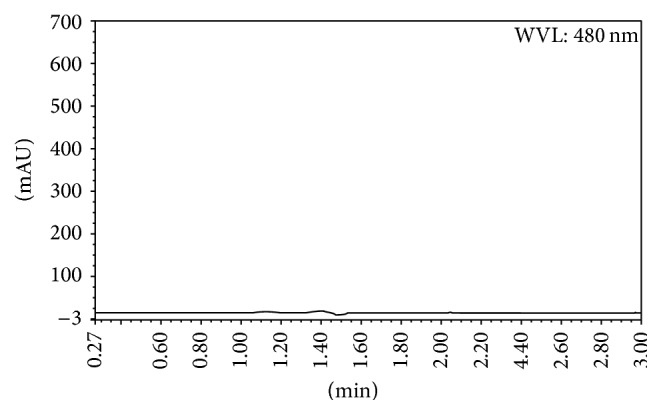
HPLC chromatograms obtained from excipients compounded from tablets.

Specificity was estimated by comparing the chromatograms of blank methanol solution, idebenone standard solution, and excipients solution from tablets as per assay method (Figure 3). The results showed that there was no interference at the retention time of idebenone from the formulation tablet components.

#### 3.2.2. Linearity

The linearity range of idebenone was demonstrated in the interval of 3.0 to 8.0 mg/mL. The mean linear regression equation obtained was *y* = 13.093*x* + 0.581, where *y* is the peak area and *x* is the standard solution concentration in mg/mL. The correlation coefficient (*r* ^2^ = 0.9987) is suitable (Table 1) to determine idebenone concentration range of 3.0 to 8.0 mg/mL.

#### 3.2.3. Limit of Detection (LOD) and Limit of Quantification (LOQ)

LOD and LOQ were 0.031 and 0.047 mg/mL, respectively.

#### 3.2.4. Precision

The results obtained for the intraday and interday precision are shown in Table 2. The RSD values were less than 1.0% for all concentrations tested and confirmed the suitable intraday and interday precision of the method.

#### 3.2.5. Accuracy

The mean recoveries were found to be 98.6 to 101.5% (Table 3). These results demonstrate accuracy for the determination of idebenone in pharmaceutical tablets.

#### 3.2.6. Robustness

The percent recoveries of idebenone were good under most conditions except for the flow rate at 1.3 mL/min (Table 4).

### 3.3. Forced Degradation Study

**Figure 4:**
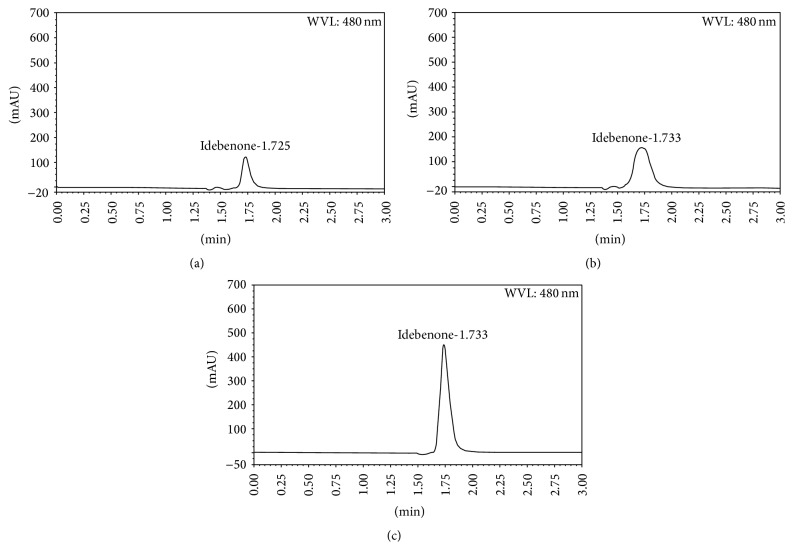
Chromatograms of 1 N HCl (a), 1 N NaOH (b), and oxidative (c) stress conditions.

As shown in Table 5, alkaline stress led to the faster effect on idebenone degradation with about 17% of idebenone remaining only after 48 h After exposure to acid stress, about 48% of idebenone was degraded showing the critical pH effects on idebenone stability (Table 5). In oxidative condition, 15% of the drug was degraded whereas in high temperature and direct sunlight conditions, idebenone was found more stable with degradation under 10% (Table 5). At ambient temperature, the drug was stable for at least 15 days. Any peak of product degradation was observed in all stress conditions (Figure 4; Table 5).

## 4. Conclusion

A simple and rapid stability-indicating high-performance liquid chromatographic method was developed and validated for the determination of idebenone in pharmaceutical dosage forms. The analytical method is specific, linear, precise, accurate, and robust for a rapid determination of this drug and can be used for studying the stability and degradation kinetics of idebenone.

## Figures and Tables

**Table 1:** Linearity data of the developed method.

Initial conc.	Mean peak area ± SD	Actual conc.	% assay
(mg/mL)	(*n* = 6)	(mg/mL)	
3.0	40.16 ± 0.08	3.02 ± 0.04	100.7
4.0	52.14 ± 0.57	3.94 ± 0.00	98.4
5.0	65.59 ± 0.28	4.96 ± 0.02	99.3
6.0	80.35 ± 0.54	6.09 ± 0.00	101.5
7.0	92.91 ± 0.25	7.05 ± 0.02	100.7
8.0	104.39 ± 0.56	7.93 ± 0.00	99.1
*y* = 13.093*x* + 0.581, *r* ^2^ = 0.9987			

**Table 2:** Precision study of the method.

Nominal conc. (mg/mL)	Intraday precision		Interday precision	
	Calculated conc. (mg/mL), mean ± SD	RSD (%)	Calculated conc. (mg/mL), mean ± SD	RSD (%)
3.5	3.46 ± 0.03	0.95	3.45 ± 0.02	0.58
4.5	4.53 ± 0.03	0.76	4.58 ± 0.03	0.69
6.5	6.51 ± 0.05	0.81	6.47 ± 0.04	0.65

**Table 3:** Accuracy of the method.

Standard (mg/mL)	Added		Found (mg/mL)	% recovery	RSD
	%	mg/mL	Mean ± SD, *n* = 6	Mean	
4.0	20	4.8	4.86 ± 0.02	101.45	0.43
4.0	40	5.6	5.52 ± 0.03	98.67	0.55
4.0	80	7.2	7.16 ± 0.04	99.52	0.52
4.0	100	8.0	8.01 ± 0.03	100.11	0.41
4.0	120	8.6	8.62 ± 0.03	100.34	0.34

**Table 4:** Robustness.

Parameters	Modification	RT (min)	% recovery
Flow rate (mL/min)	1.1	1.558	99.9
	1.2	1.425	91.2
	1.3	1.317	85.2

Wavelength of detection (nm)	481	1.71	101.7
	482	1.70	101.3
	483	1.70	98.2

Column temperature (°C)	23	1.72	100.7
	27	1.70	100.3
	30	1.69	100.6

Deionised water in mobile phase	1%	1.71	100.9
	2%	1.75	101.9

**Table 5:** Forced degradation study.

Stress conditions	% remaining	% degradation	Retention time of degraded products^a^
Acidic stress (1N HCl, 50°C, 1 h)	51.8	48.2	—
Alkaline stress (1N NaOH, 50°C, 1 h)	16.6	83.4	—
Oxidative stress (3%, 50°C, 48 h)	84.6	15.4	—
Thermal stress (50°C, 8 days)	99.3	0.7	—
Direct sunlight (8 days)	95.8	4.2	—
Ambient temperature (15 days)	92.0	8.0	—

^a^No peak detected.

## References

[1] Esposti M. D., Ngo A., Ghelli A. (1996). The interaction of Q analogs, particularly hydroxydecyl benzoquinone (idebenone), with the respiratory complexes of heart mitochondria. *Archives of Biochemistry and Biophysics*.

[2] Geromel V., Darin N., Chrétien D. (2002). Coenzyme Q10 and idebenone in the therapy of respiratory chain diseases: rationale and comparative benefits. *Molecular Genetics and Metabolism*.

[3] Nagaoka A., Suno M., Shibota M., Kakihana M. (1989). Effects of idebenone on neurological deficits, local cerebral blood flow, and energy metabolism in rats with experimental cerebral ischemia. *Archives of Gerontology and Geriatrics*.

[4] Zs.-Nagy I. (1990). Chemistry, toxicology, pharmacology and pharmacokinetics of idebenone: a review. *Archives of Gerontology and Geriatrics*.

[5] Mercuri E., Muntoni F. (2015). Efficacy of idebenone in Duchenne muscular dystrophy. *The Lancet*.

[6] Klopstock T., Metz G., Yu-Wai-Man P. (2013). Persistence of the treatment effect of idebenone in Leber's hereditary optic neuropathy. *Brain*.

[7] Parkinson M. H., Schulz J. B., Giunti P. (2013). Co-enzyme Q10 and idebenone use in Friedreich's ataxia. *Journal of Neurochemistry*.

[8] Lang J. K., Gohil K., Packer L. (1986). Simultaneous determination of tocopherols, ubiquinols, and ubiquinones in blood, plasma, tissue homogenates, and subcellular fractions. *Analytical Biochemistry*.

[9] Lang J. K., Packer L. (1987). Quantitative determination of vitamin E and oxidized and reduced coenzyme Q by high-performance liquid chromatography with in-line ultraviolet and electrochemical detection. *Journal of Chromatography A*.

[10] ICH Q2(R1) validation of analytical procedures: text and methodology. http://www.ich.org/fileadmin/Public_Web_Site/ICH_Products/Guidelines/Quality/Q2_R1/Step4/Q2_R1__Guideline.pdf.

